# Supplementary material for: Polar discontinuities, emergent conductivity, and critical twist-angle-dependent behaviour at wafer-bonded ferroelectric interfaces
Source: Nat Commun. 2026 Jan 23;17:1842. doi: 10.1038/s41467-026-68553-7 (PMC12921239; doi:10.1038/s41467-026-68553-7)
Supplement: Supplementary file 1 — Supplementary Information [file 41467_2026_68553_MOESM1_ESM.pdf]

supplementary information for

Polar Discontinuities, Emergent Conductivity, and Critical Twist-Angle-Dependent Behaviour at Wafer-Bonded Ferroelectric Interfaces

Andrew Rogers†, Kristina Holsgrove*†, Nils A. Schäfer, Boris Koppitz, Conor J. McCluskey, Shivani Yedama, Ronan Lynch, Keelan Sloan, Barry Porter, Adam Sykes, Alex Catalan Daniels, Romualdo S. Silva Jr, Flavio Y. Bruno, Sam D. Seddon, Haidong Lu, Michael Ruesing, Christa Fink, Philipp Fahler-Muenzer, Sarah Fearn, Sandrine E. M. Heutz, Marios Hadjimichael, Quentin M. Ramasse, Marin Alexe, Amit Kumar, Raymond G. P. McQuaid, Alexei Gruverman, Simone Sanna, Lukas M. Eng, J. Marty Gregg#

† These authors contributed equally to this work.
Corresponding authors: #m.gregg@qub.ac.uk and *kholsgrove04@qub.ac.uk

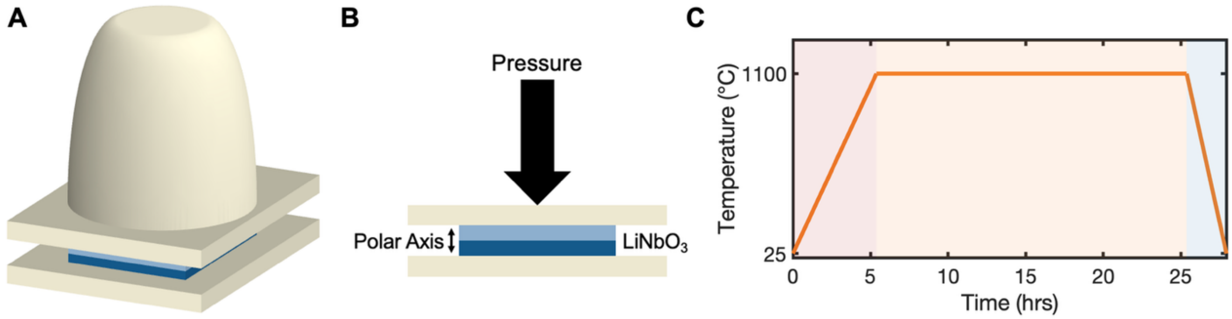

Figure S1. Thermocompression bonding process. (**A-B**) Two pieces of single crystal z-cut Lithium Niobate ($LiNbO_3$) are placed in a furnace with a 500g zirconium oxide weight placed on top. (**C**) Furnace heating cycle. The crystals are heated from room temperature to 1100°C at a rate of 3.33°C/min. They are held at 1100°C for 20 hours, before being cooled back to room temperature at a rate of 7°C/min*.

**Note – Our furnace does not have active cooling, and therefore whilst it is programmed to cool at 7°C/min, in reality it cools non-linearly and at a far slower rate. At high temperatures this rate of cooling is a close approximation, but for the mid-to-low temperature range the cooling takes place substantially more slowly.*

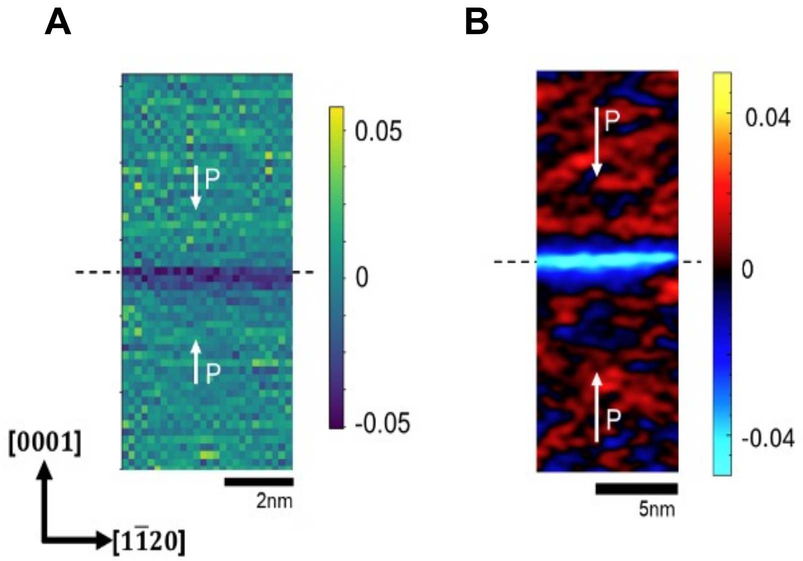

Figure S2. Confirmation of Nb-Nb Compression at the Bonded Interface. Two additional techniques, beyond those illustrated in the figures of the main manuscript, have been used to ensure that the claimed reduction in Nb-Nb interatomic separation, along the [0001] direction, at the interface is robust. In (A) Atomap (a python package for identifying atomic columns and measuring distances between them – see https://atomap.org/) has been used to identify the Nb column positions and variations in interatomic positions – note the distinct dark band at the interface in which relative separations are distinctly reduced. Here the scale bar indicates the colours associated with different fractional variations of interatomic separations relative to a reference (representative of bulk). In (B), conventional Geometric Phase Analysis (GPA) has been used. This image shows ε_{yy} where y is the direction parallel and antiparallel to the polarisation directions in the two crystals (marked by white arrows). Again, distinct compression in interatomic separations along the [0001] can be seen. The scale bar shows colours associated with differing values for the ε_{yy} components in the 2D strain tensor, calculated with respect to a unit mesh from the bulk region of the LNO.

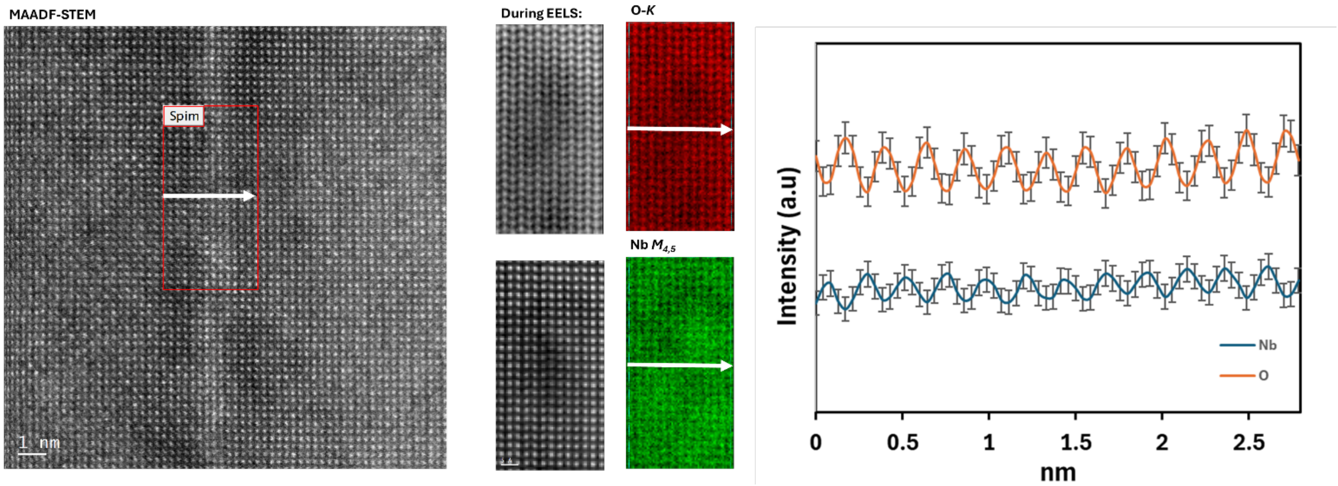

Figure S3.1. MAADF-STEM and EELS data for untwisted H2H sample. Line profile averaged across entire O-*K* map and Nb $M_{4,5}$ map (in direction of arrow) reveals homogeneous (within error) concentration of O and Nb transitioning from one crystal (across interface) to the other crystal. Area corresponds to Figure 3 in main paper.

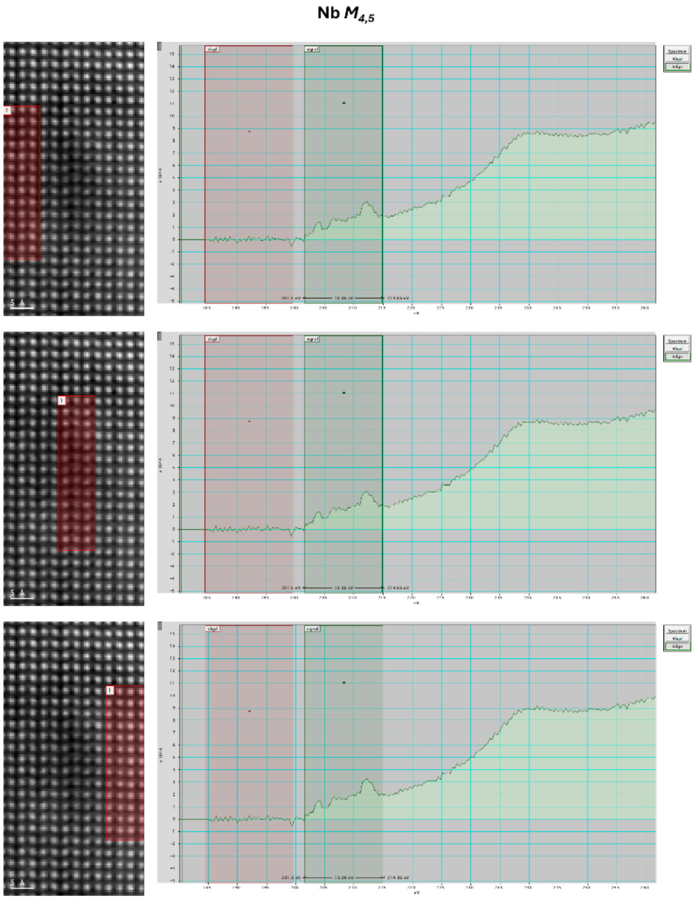

Figure S3.2. Raw EELS spectra for Nb M4,5 edge transitioning across the interface. No obvious change in edge shape. Area corresponds to Figure 3 in main paper.

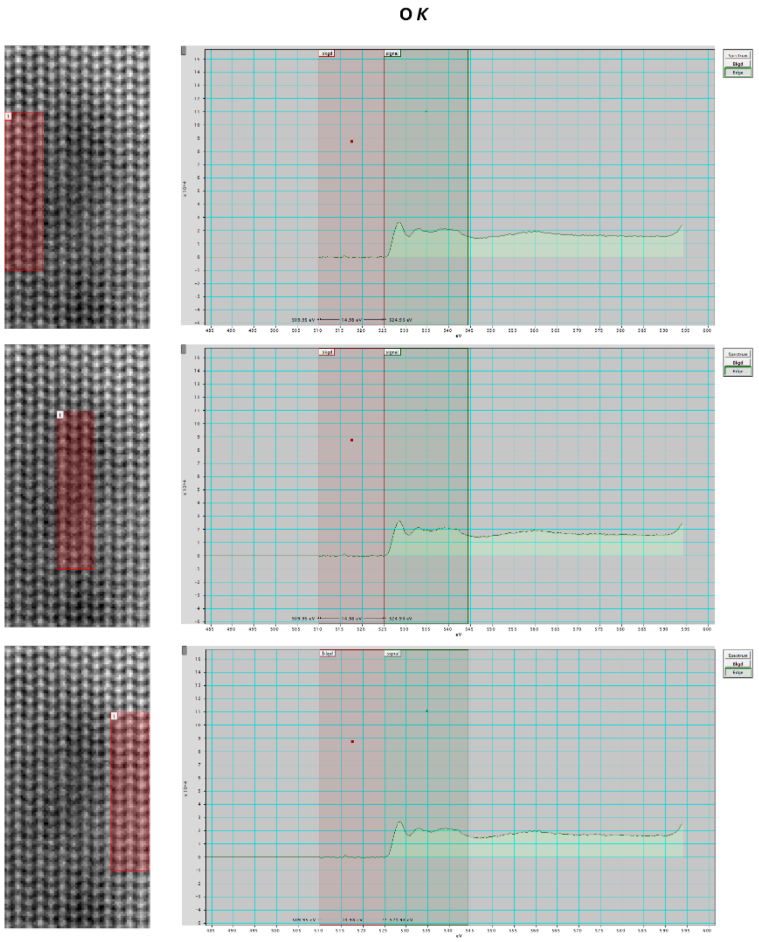

Figure S3.3. Raw EELS spectra for O *K* edge transitioning across the interface. No obvious change in edge shape. Area corresponds to Figure 3 in main paper.

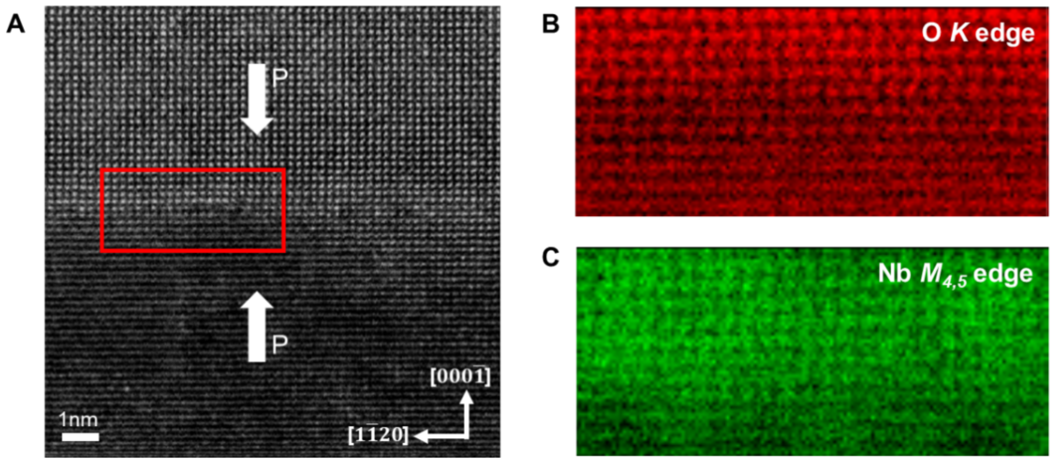

Figure S4. HAADF-STEM and EELS data for untwisted H2H sample (different interface area). (A) HAADF-STEM image and EELS signal map for (B) O *K* edge and (C) Nb $M_{4,5}$ edge on a region of the H-H interface where the bottom crystal is slightly misoriented in terms of the electron beam direction. Homogeneous (within error) concentration of O and Nb prevail.

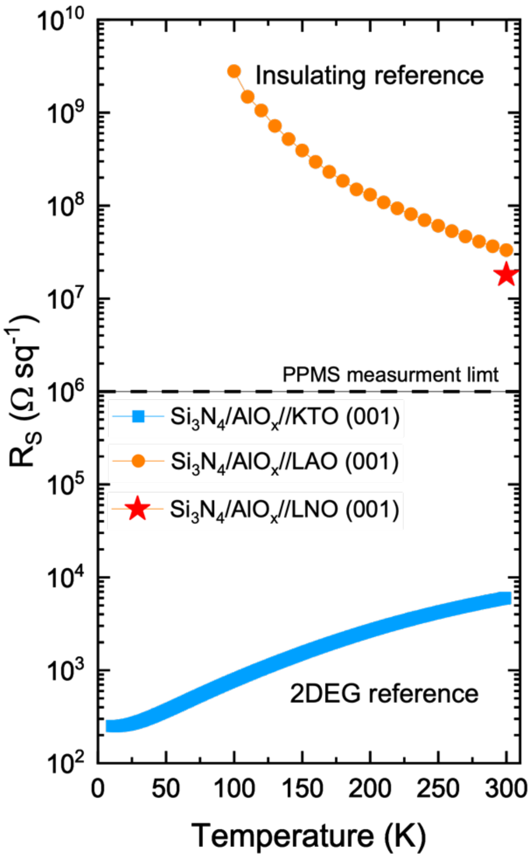

Figure S5. Sheet resistance as a function of temperature. for reference samples showing a metallic 2DEG (blue-$KTaO_3$ substrate), and insulating behaviour (orange – $LaAlO_3$ substrate). The red star displays the room temperature sheet resistance of the Si_3N_4/AlO_x bilayer grown on $LiNbO_3$.

Fig. S5 Further Experimental Details

We fabricated Si_3N_4/AlOx bilayers on $LiNbO_3$, $KTaO_3$, and $LaAlO_3$ substrates following the procedure described in [1]. The Al layer was deposited under a pure Ar atmosphere and subsequently oxidized into AlO_x via a redox reaction that extracts oxygen from the underlying substrate, creating oxygen vacancies. In materials such as $SrTiO_3$ and $KTaO_3$, this process is known to induce a conductive two-dimensional electron gas (2DEG). In contrast, substrates like $LaAlO_3$ remain insulating under identical conditions.

To characterize the transport properties, resistance as a function of temperature was measured using a Quantum Design PPMS system. All low-noise measurements were performed

in a four-probe configuration, using a current of 10 μA and a power limit of 100 μW, which sets an upper sheet resistance (Rs) detection limit of approximately 1 MΩ. For more resistive samples, Rs at room temperature was also measured using a Keithley 2450 Source Measure Unit (SMU) in a four-point probe station. In the case of the Si_3N_4/AlOx /$LaAlO_3$ sample, the resistance exceeded the PPMS measurement range. Therefore, a Keithley SMU was externally connected to the PPMS system to extend the dynamic range.

As previously discussed [1], $LaAlO_3$ itself remains insulating, while the capping Si_3N_4 layer can exhibit residual semiconducting behaviour due to nitrogen vacancies. Similarly, the sample grown on $LiNbO_3$ was too resistive to be measured within the PPMS limits. The room-temperature Rs measured with the Keithley SMU was comparable to that of the $LaAlO_3$-based sample, indicating the absence of a conductive 2DEG and suggesting that any residual conductivity originates from the capping layer rather than from the substrate interface.

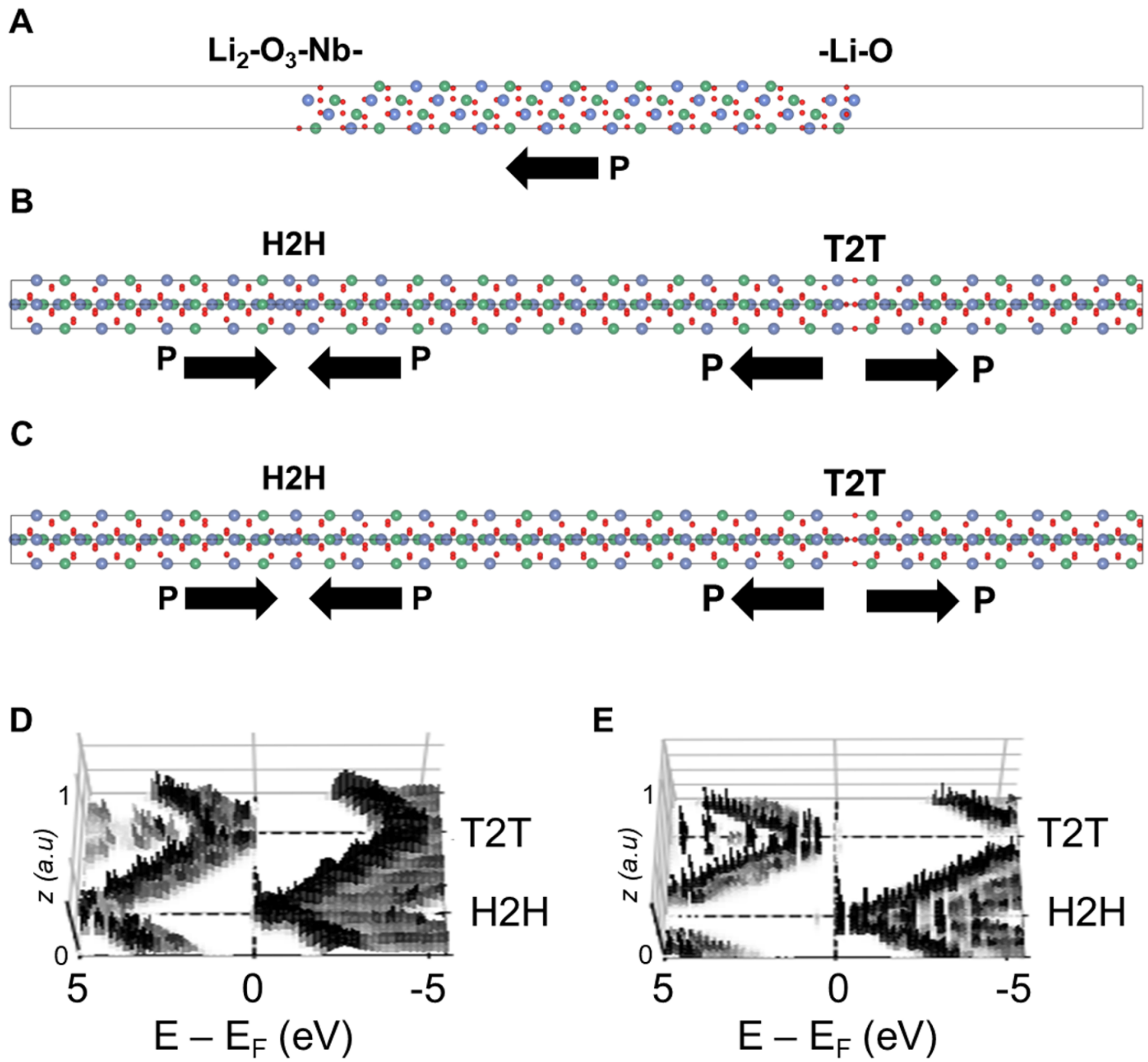

Figure S6. Modelled $LiNbO_3$ structure. (A) Thin film of $LiNbO_3$ consisting of 128 atoms with the thermodynamic stable terminations, where Li is shown in blue, Nb in green, and O in red. (B) Bonded structure formed by combining two thin films to create both a H2H and T2T interface for a 0 ° twist and (C) a 60 ° twist. Viewing direction $[1\bar{1}00]$. (D) Real space representation of the calculated DOS for 60 ° twisted thin films along the c-axis and (E) for 0 ° in the HSE06 approximation.

Fig. S6 Further Computational Details

To explore the bonding characteristics of *z*-cut $LiNbO_3$ thin films, first-principles calculations were carried out using DFT as implemented in VASP [2–4]. The projector-augmented wave (PAW) method [5,6], along with the PBEsol functional [7] is applied. The cutoff for the plane wave basis is

set at 475 eV. Explicit treatment is given to 1, 13, and 6 valence electrons for Li, Nb, and O, respectively. Structural relaxations are performed using the RMM-DIIS algorithm [8] until the forces acting on the single atoms are smaller than 0.005 eV/Å. The convergence criterion for the self-consistent determination of the electronic charge is reached if total energy changes between consecutive iterations fall below 10^{-6} eV. Thereby the energy integration is performed utilising a Γ centred 4 x 4 x 1 k-point mesh. The computational approach is thus on the same footing of atomistic calculations which have been successfully employed to model H2H or T2T geometries of different ferroelectric materials.

Initially, the thermodynamically stable surfaces of z-cut $LiNbO_3$ are recreated using a supercell containing 128 atoms and a vacuum region of 30 Å, as described in [9]. This geometry is selected to ensure that the separation between two CDW, as determined in [10], is large enough to minimise their interaction. The lattice parameters of the hexagonal unit cell (a = 5.13 Å, c = 13.80 Å) are derived from a PBEsol calculation and used to construct the thin films. The single surfaces are terminated with O-Li- at the tail and -Nb-O_3-Li_2 at the head. Structural relaxations are performed only for the first 20 atoms at the positive surface and the first 17 atoms at the negative surface of the thin film, while keeping the other atoms fixed at the bulk positions. Subsequently, two thin films are aligned atop one another to model both the H2H and T2T configurations simultaneously. The separation between the films is allowed to relax in a manner consistent with that of a single thin film. Surface separation distances are systematically sampled and analysed by fitting the data to the Murnaghan equation of state [11,12]. The interface region is allowed to relax in a manner consistent with that of a single thin film (Hellmann-Feynman force threshold of 0.005 eV/Å), in order to model a structurally optimised interface.

Once the optimal spacing between the films is established, a vacuum layer as large as 30 Å is introduced between the thin films to facilitate surface energy calculations while modelling a single H2H or a single T2D CDW. Additionally, the projected band structure, DOS, and the spatial distribution of the free charge carriers within 0.1 eV of the Fermi energy are computed. The total free charge carriers present are 4.091 x 10^{21} e/cm^3 and 4.563 x 10^{21} e/cm^3 for the H2H and T2T interfaces, respectively. Note that the DOS retains its general shape when using the HSE06 functional [13,14], aside from a larger band gap

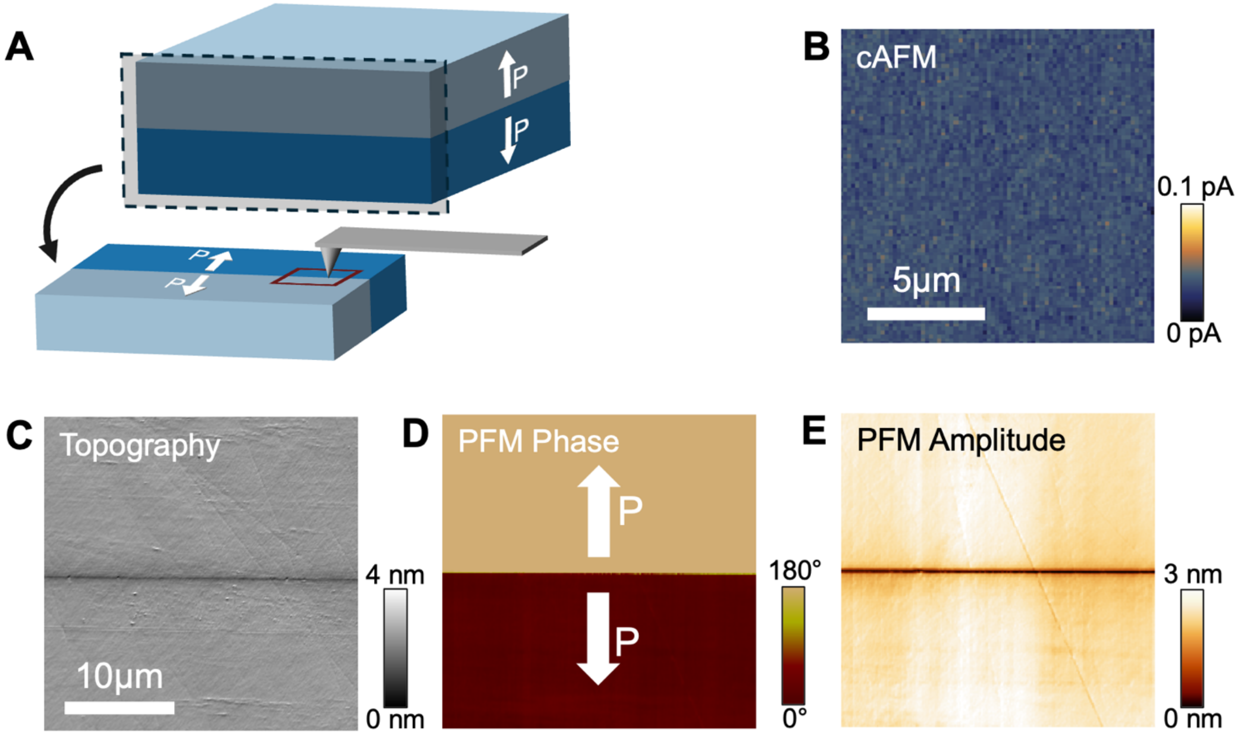

Figure S7. Tail-To-Tail (T2T) Sample Without Deliberate Twist. (A) Schematic of sample processing post-annealing. The sample is cut with a diamond saw along the dashed plane, rotated to place the interface in the vertical direction, and the top face is polished. (B) cAFM on T2T interface with applied bias of -9.5V. No cAFM contrast associated with current flow at the interface is observed. (C) AFM topography. Small decrease in height at the interface due to preferential etching during polishing. (D) PFM phase. (E) PFM amplitude.

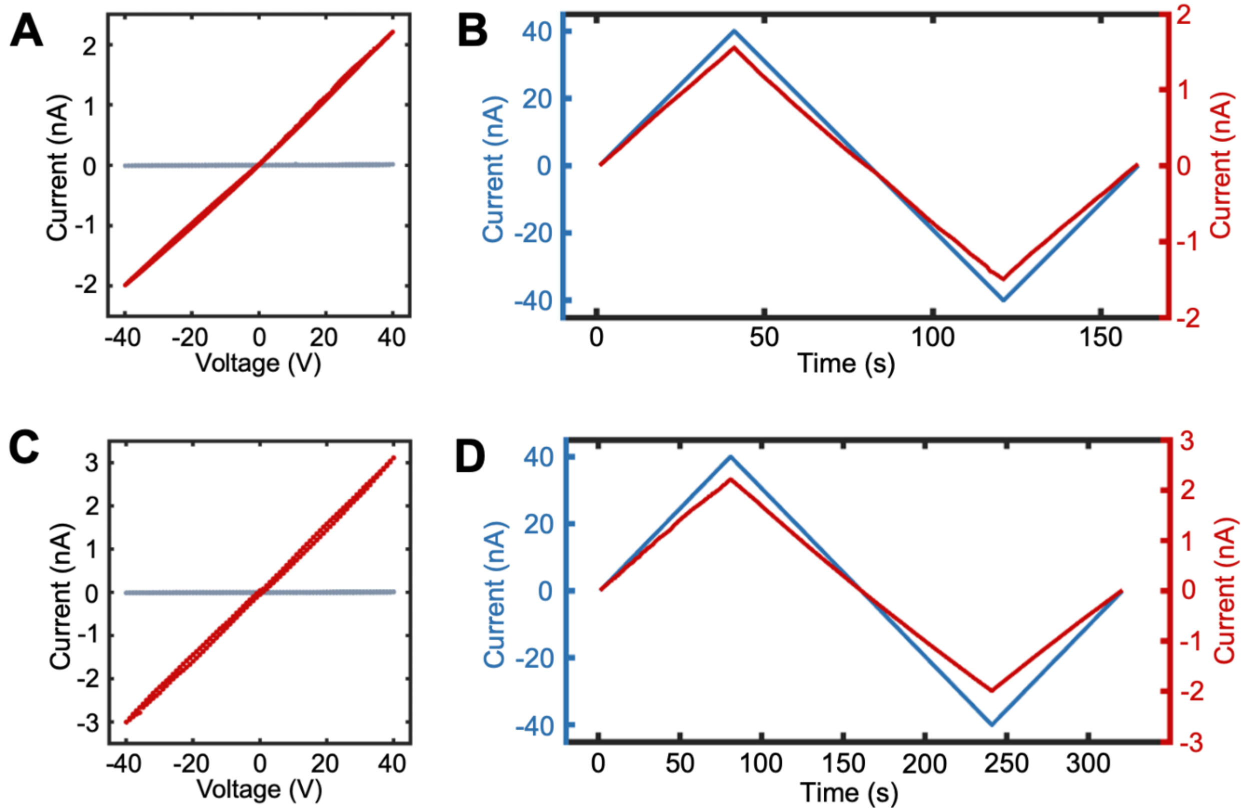

Figure S8. IV data for ~0° H2H and T2T samples. (A) IV plot for ~0° H2H sample. Bulk LNO plotted in grey. (B) IV characteristics of ~0° H2H sample with respect to time. (C) IV plot for ~0° T2T sample. Bulk LNO plotted in grey. (D) IV characteristics of ~0° T2T sample with respect to time. As can be seen, similar levels of conduction are associated with the two interface types. However, T2T interfaces have not been seen to develop distinct cAFM signatures. We expect that the conduction at T2T interfaces is more diffuse than at H2H ones.

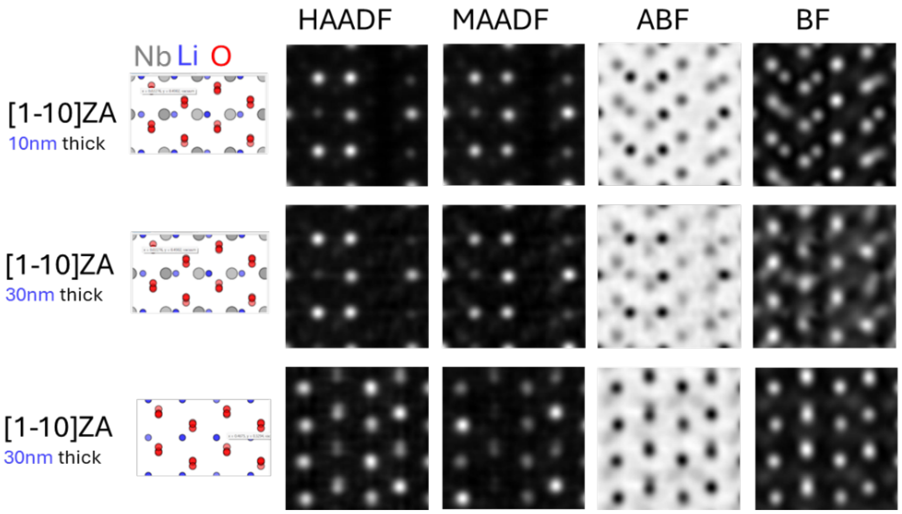

Figure S9. Simulations performed using Dr. Probe utilising the STEM detector collection angles experimentally used at SuperSTEM. The $LiNbO_3$ unit cell for each set of simulations is shown on the left (viewed down [1-100] zone axis). It is apparent that altering the specimen thickness from 10nm to 30nm (more comparable to the real TEM specimen) has little change on the STEM images produced: the HAADF and MAADF images display the heaviest atom, Nb, while the ABF and BF images display both the heaviest atom, Nb, and the next heaviest atom, O. The Li atoms are not observed in any detector image. However, when the input unit cell was altered so that the heaviest atoms, Nb, were artificially deleted (bottom row of images) then there was a faint trace of the lightest atoms, Li in the resultant ABF and BF images. We can conclude from these simulations that when Nb is present (which is reflective of the genuine unit cell of $LiNbO_3$) it is not possible to visualise nor identify the exact location of Li ion columns.

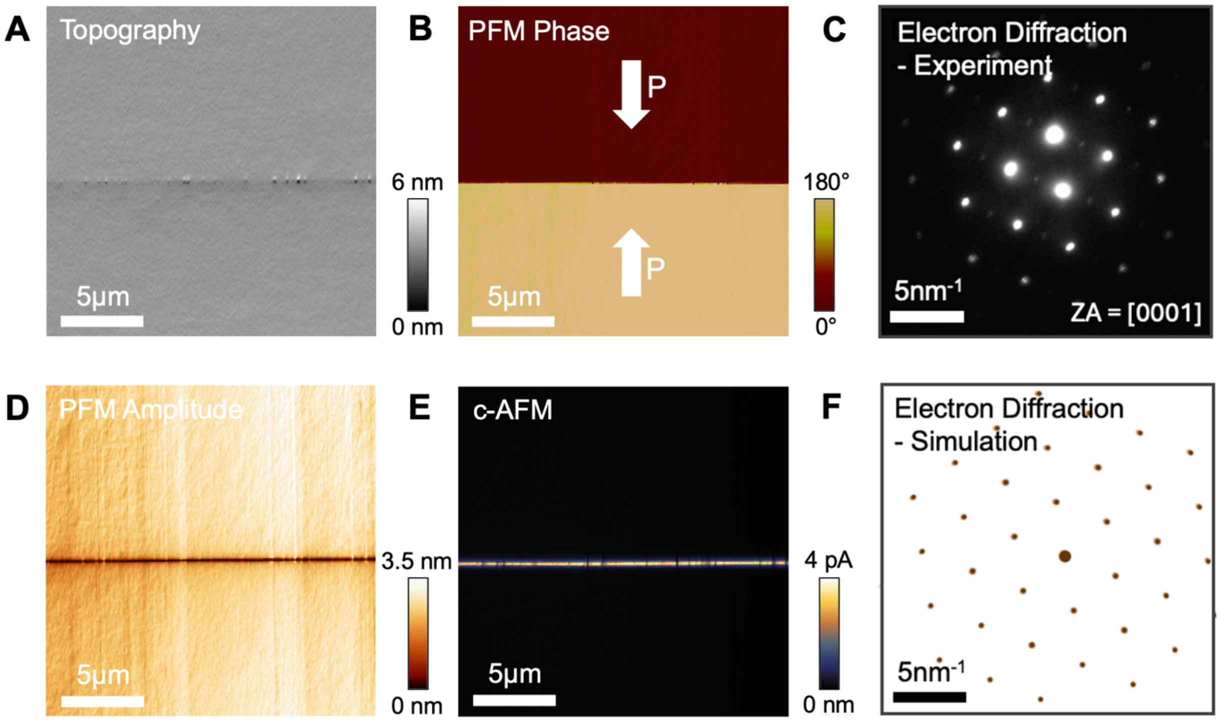

Figure S10. Atomic Force Microscopy and Electron Diffraction data for H2H bonded sample with ~60° of deliberate twist. (A) Topography (B) PFM Phase (C) TEM electron diffraction at the twisted interface, taken along the zone axis [0001]. (D) PFM amplitude. (E) cAFM with -9.5V bias applied to the base of the sample. Results look to mirror those for H2H samples without any deliberate relative twist in the two bonded crystals. (F) Simulated electron diffraction for an interfacial lattice twist of 60°.

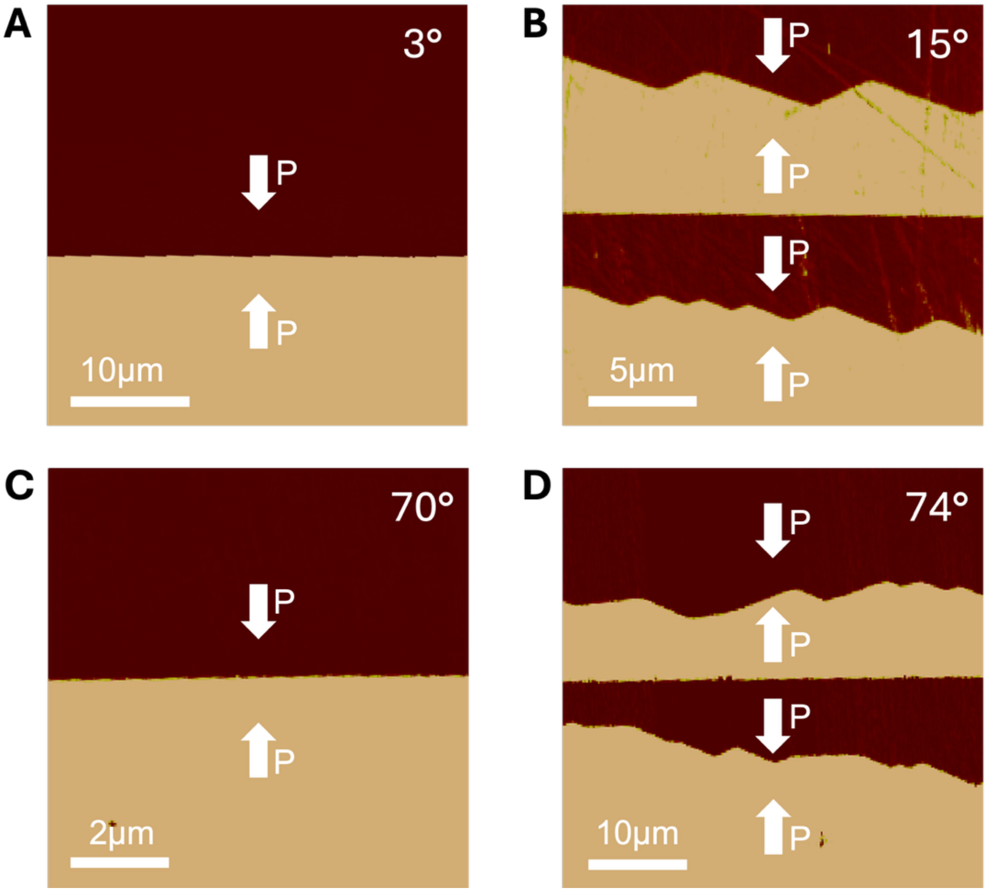

Figure S11. Piezoresponse Force Microscopy (PFM) data for interfaces set originally as H2H with various twist angles. Samples were all put into the furnace in H2H configurations. (A) The lateral PFM phase map after bonding with ~3° twist. Clearly, the H2H polar discontinuity is maintained. (B) With ~15° relative twist, interfacial domain inversion is observed. (C) With ~70° of twist, the H2H polar discontinuity is maintained, while in (D) a ~74° twist again produces interfacial domain inversion. We suspect the inversion phenomenon is associated with a shutting down of the interface conductivity in the H2H bonds (as this microstructural rearrangement is typically seen at c+ faces of LNO crystals when heat treated without deliberate charge screening). We note that the twist angles concerned are, within reasonable twist uncertainty, of those at which anomalies in the moiré periodicity as a function of twist angle occur.

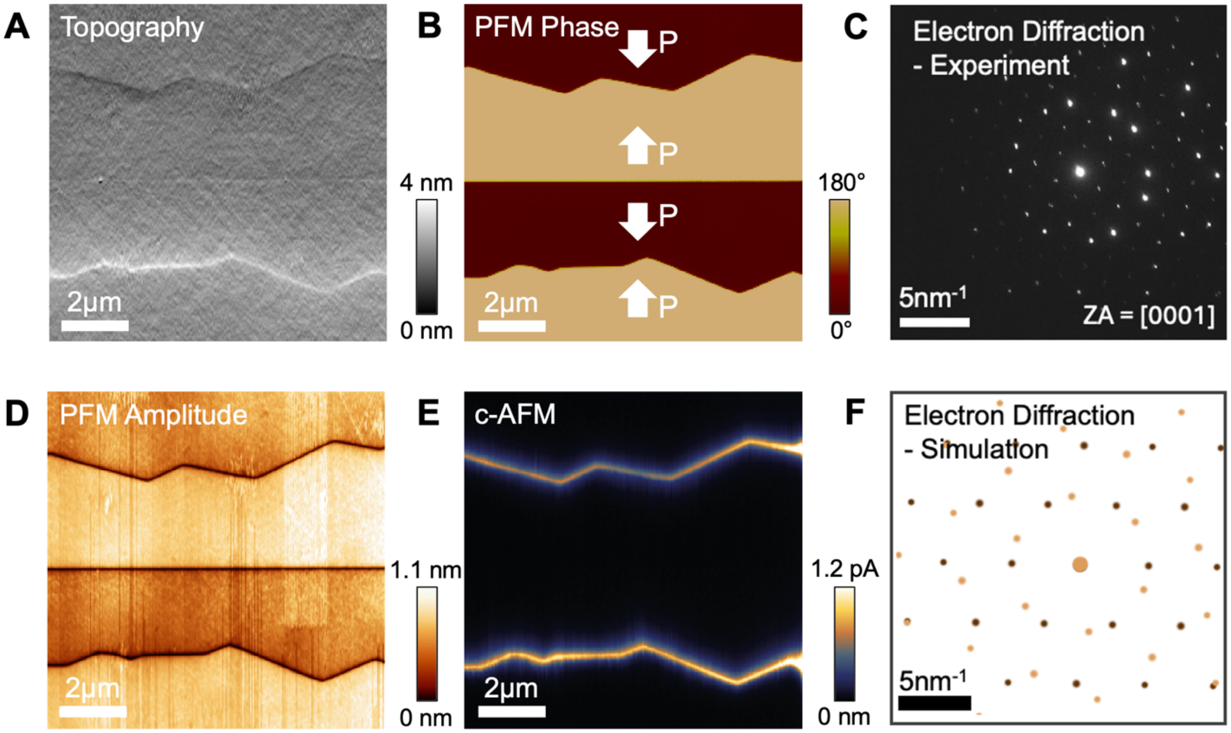

Figure S12. Atomic Force Microscopy and Electron Diffraction data for a sample with ~21° in relative twist. (A) Topography (B) PFM Phase (C) TEM electron diffraction at the twisted interface, taken along the zone axis [0001]. (D) PFM amplitude. (E) cAFM with -9.5V bias applied to the base of the sample. The inversion in the orientation of the polarization at the interface is evident. (F) Simulated electron diffraction for an interfacial lattice twist of 21°.

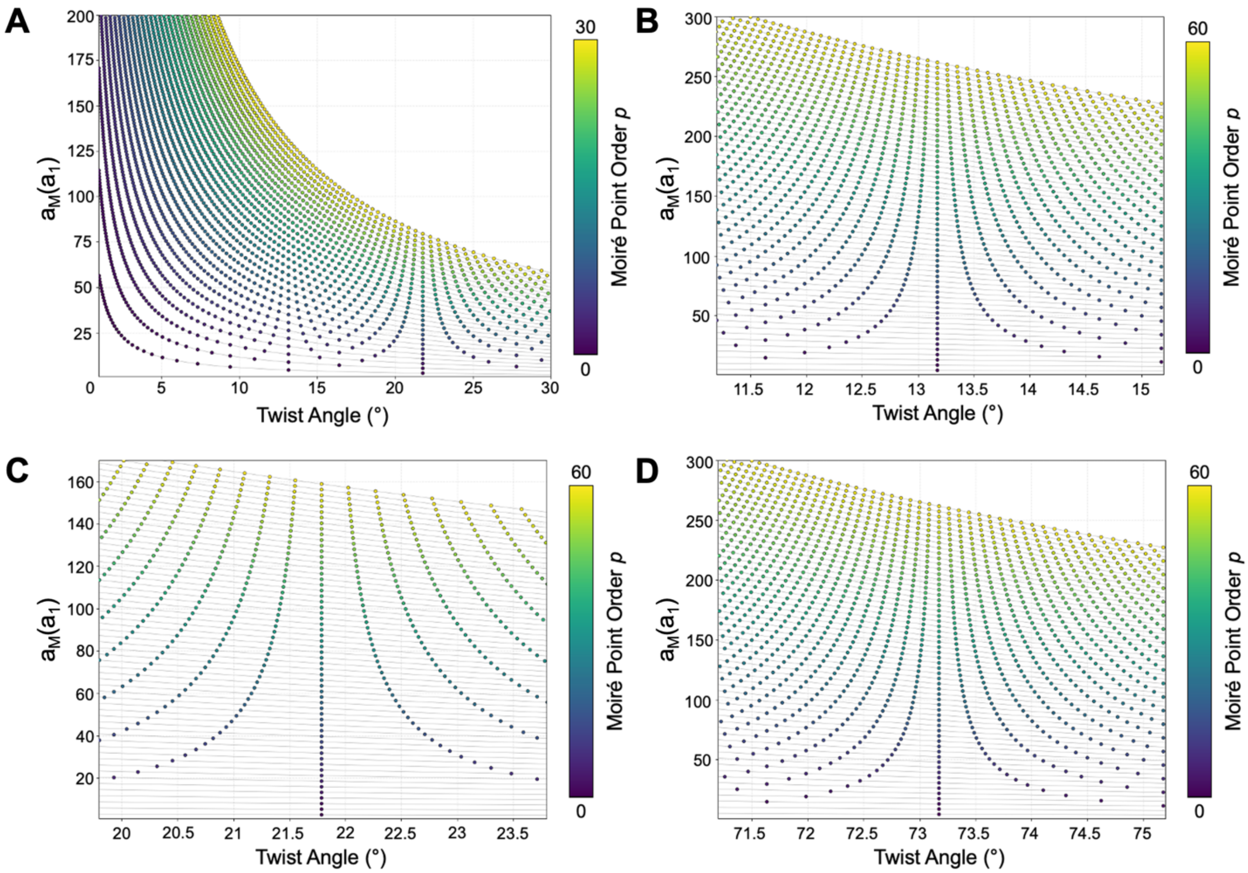

Figure S13. Plots made using approach used in reference [15]. Here, coincident lattice periods are presented (as dots) that occur when two trigonal (hexagonal) lattices, sharing {0001} interfaces, are twisted about <0001> axes. Note that the data points are distinct solutions. In all plots (A-D), the coincident lattice periodicity (a_M) is given relative to that of the fundamental lattice (a_1). Note the paucity of exact coincident lattice solutions close to angles of 13.2°, 21.8° and 73.2° and the fact that polar inversions, at $LiNbO_3$ bonded interfaces, were only observed in our work at twist angles of ~14°, ~21°, and ~74°. We suspect that our inferred twist-induced collapse in the conductivity at the bonded interface might be associated with the extremely large coincident lattice spacings found at these twist angles.

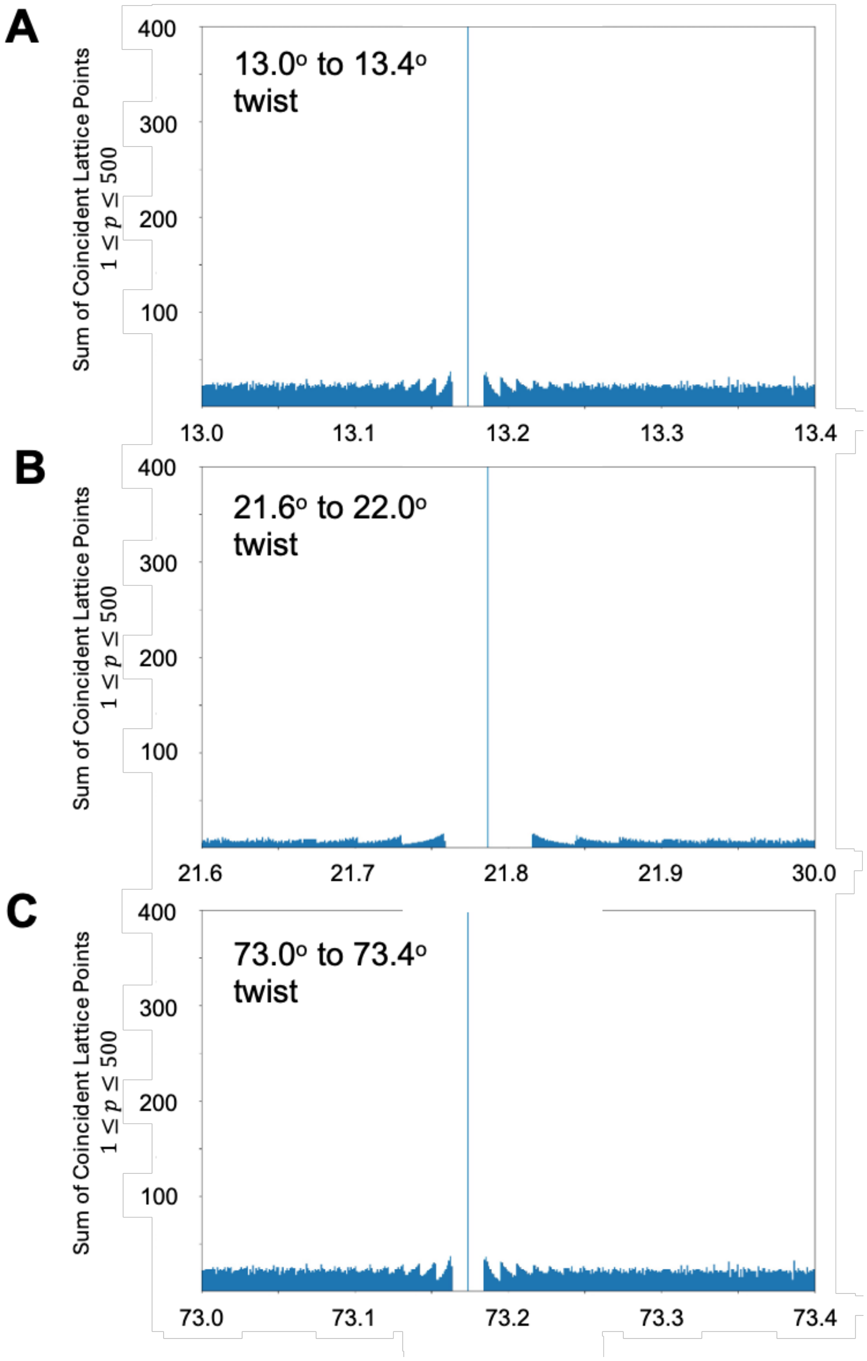

Figure S14: Coincident lattice density at interfaces for specific ranges in twist angle. Information derived from (*13*) and from plots of the kind shown in figure S11. The total number of coincident lattice points as a function of twist angle (summed within an angular range of 0.001º around each twist angle), when the parameter "*p*" (as defined in (*15*)) is varied from 1 to 500. Note the distinct "band gaps", where no coincident lattices occur, found between ~13.16º and 13.18º (**A**), ~21.76º and ~21.82º (**B**) and ~73.16º and ~73.18º (**C**).

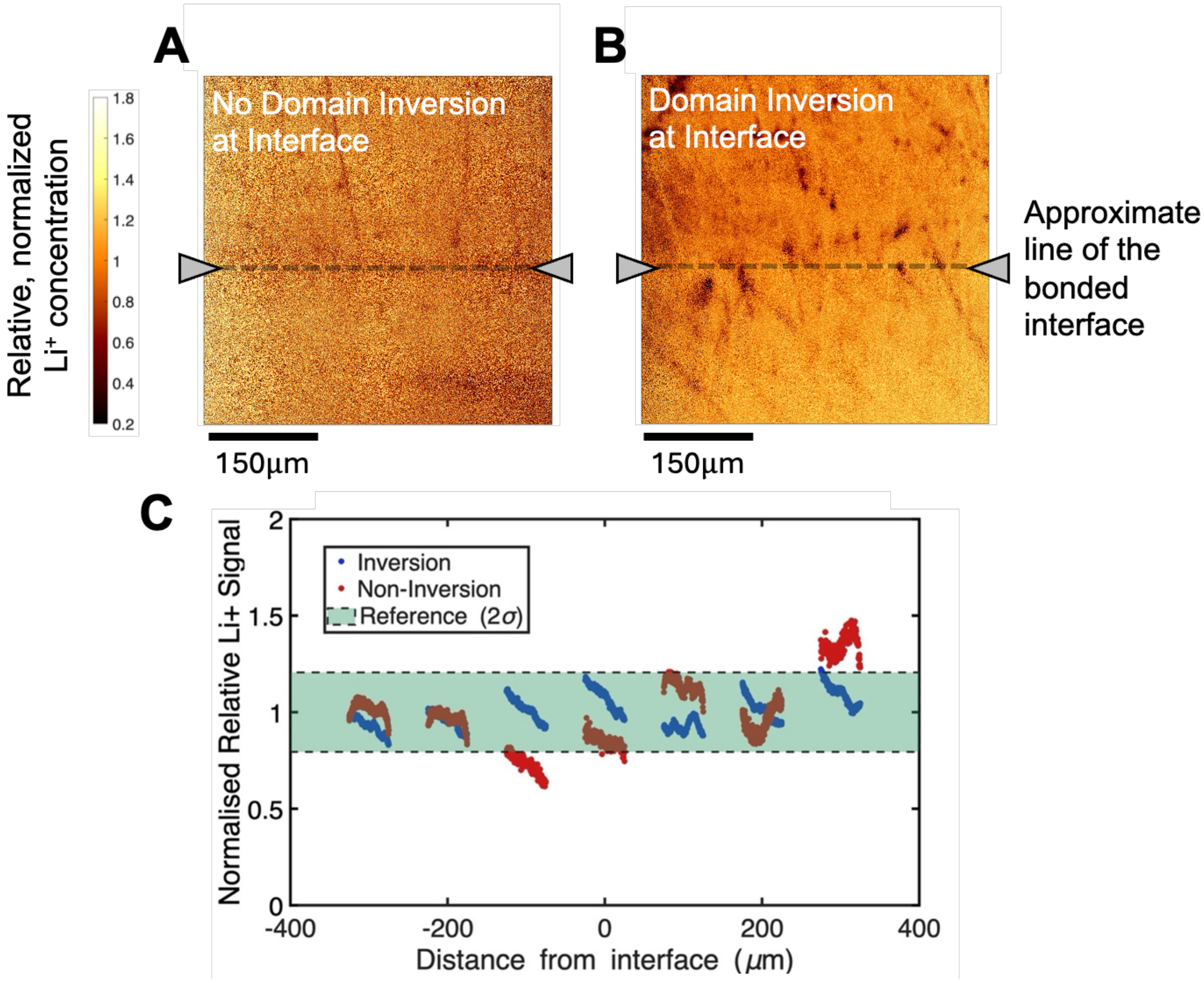

Figure S15: Lithium concentration as a function of distance from the bonded interfaces. Secondary ion mass spectroscopy imaging of the bonded head-to-head $LiNbO_3$ interfaces: Spatially resolved maps of the normalised, relative Li+ concentrations in the head-to-head bonded samples that maintained their H2H configuration (**A**) and in those in which a local polar inversion occurred adjacent to the interface to change it to a T2T configuration (**B**). The black dotted line indicates the approximate location of the bonded interface, which runs horizontally. (**C**) Li+ concentration profiles, comprised of multiple 50µm x 50µm SIMS images taken on a trace running perpendicular to the interface, for both the non-inverted and inverted samples. The variation of Li+ across a reference $LiNbO_3$ single crystal, which was not subjected to heat treatment, is shown by the green shaded area.

References

1. Martínez, E. A. *et al.* Synthesis and in-depth interfacial characterization of 2D electron gases formed in Si_3N_4/Al//$KTaO_3$ heterostructures. *Appl Surf Sci* **689**, 162499 (2025).
2. Kresse, G. & Hafner, J. *Ab initio* molecular dynamics for liquid metals. *Phys Rev B* **47**, 558–561 (1993).
3. Kresse, G. & Furthmüller, J. Efficient iterative schemes for *ab initio* total-energy calculations using a plane-wave basis set. *Phys Rev B* **54**, 11169–11186 (1996).
4. Kresse, G. & Furthmüller, J. Efficiency of ab-initio total energy calculations for metals and semiconductors using a plane-wave basis set. *Comput Mater Sci* **6**, 15–50 (1996).
5. Kresse, G. & Joubert, D. From ultrasoft pseudopotentials to the projector augmented-wave method. *Phys Rev B* **59**, 1758–1775 (1999).
6. Blöchl, P. E. Projector augmented-wave method. *Phys Rev B* **50**, 17953–17979 (1994).
7. Perdew, J. P. *et al.* Restoring the Density-Gradient Expansion for Exchange in Solids and Surfaces. *Phys Rev Lett* **100**, 136406 (2008).
8. Pulay, P. Convergence acceleration of iterative sequences. the case of scf iteration. *Chem Phys Lett* **73**, 393–398 (1980).
9. Sanna, S. & Schmidt, W. G. $LiNbO_3$ surfaces from a microscopic perspective. *Journal of Physics: Condensed Matter* **29**, 413001 (2017).
10. Verhoff, L. M. *et al.* Two-dimensional electronic conductivity in insulating ferroelectrics: Peculiar properties of domain walls. *Phys Rev Res* **6**, (2024).
11. Murnaghan, F. D. The Compressibility of Media under Extreme Pressures. *Proceedings of the National Academy of Sciences* **30**, 244–247 (1944).
12. Fu, C.-L. & Ho, K.-M. First-principles calculation of the equilibrium ground-state properties of transition metals: Applications to Nb and Mo. *Phys Rev B* **28**, 5480–5486 (1983).
13. Heyd, J., Scuseria, G. E. & Ernzerhof, M. Hybrid functionals based on a screened Coulomb potential. *J Chem Phys* **118**, 8207–8215 (2003).
14. Heyd, J., Scuseria, G. E. & Ernzerhof, M. Erratum: "Hybrid functionals based on a screened Coulomb potential" [J. Chem. Phys. 118, 8207 (2003)]. *J Chem Phys* **124**, (2006).
15. Feuerbacher, M. Moiré, Euler and self-similarity – the lattice parameters of twisted hexagonal crystals. *Acta Crystallogr A Found Adv* **77**, 460–471 (2021).
